# Risk factors for adverse events following rabies vaccination: a multivariate analysis of real-world data

**DOI:** 10.3389/fpubh.2026.1721524

**Published:** 2026-02-04

**Authors:** Hao Xu, Wenjie Yan, Qiye Ma

**Affiliations:** 1 Department of Emergency, Ningbo Ninth Hospital, Ningbo, China; 2 Department of Integrated Traditional Chinese and Western Medicine, Ningbo Ninth Hospital, Ningbo, China

**Keywords:** bilateral deltoid vaccination, nomogram prediction model, rabies vaccine adverse reactions, real-world data, risk stratification

## Abstract

**Purpose:**

To investigate independent risk and protective factors associated with adverse reactions following rabies vaccination in a Chinese population; to develop and validate a predictive nomogram for clinical utility; to support risk stratification management in clinical practice.

**Methods:**

A retrospective analysis was conducted on data from 806 patients who visited the Animal Bite Outpatient Clinic of Ningbo Ninth Hospital between September 2023 and August 2025. Variables were screened using LASSO regression, and a multivariate logistic regression-based nomogram model was constructed. The model’s performance was evaluated using receiver operating characteristic (ROC) curves, calibration curves, decision curve analysis, and ten-fold cross-validation.

**Results:**

This study identified high-risk exposure grade (OR = 1.37), bilateral deltoid vaccination (OR = 4.93), history of allergy (OR = 5.07), and delayed wound management (OR = 2.13) as significant risk factors (*p* < 0.05). In contrast, a 4-dose immunization schedule (OR < 0.01) and greater injection-site skinfold thickness (OR < 0.01) were key protective factors. The constructed nomogram demonstrated excellent discriminative ability, with AUC values of 0.826 in the training set and 0.817 in the test set. Ten-fold cross-validation yielded an accuracy of 80.0%. Decision curve analysis indicated a clinical net benefit threshold ranging from 4 to 97%. Subgroup analysis newly revealed that hypertensive patients experienced a 4.4-fold increase in risk (OR = 4.43) following high-risk exposure. Additionally, vaccination during summer and autumn was associated with significantly elevated risks (OR range: 2.11–3.74).

**Conclusion:**

This study is the first to identify bilateral deltoid vaccination as an independent risk factor for adverse reactions to rabies vaccine. The constructed nomogram model demonstrates high accuracy in identifying high-risk individuals. Clinically, it is recommended that high-risk exposure patients receive single-site vaccination combined with a 4-dose regimen, with wound management completed within 24 h. Enhanced monitoring is advised for hypertensive patients to mitigate potential risks.

## Introduction

Rabies is an almost 100% fatal zoonotic disease (1, 2). Post-exposure prophylaxis (PEP), including vaccination, remains the most effective means of preventing clinical onset (3, 4). However, once neurological symptoms appear, there are no effective treatments (5). Mainland China administers a massive number of rabies vaccinations (6). Although animal rabies surveillance data (2004–2018) provide a scientific basis for vaccine allocation (7), adverse reactions to vaccination continue to lead to increased treatment discontinuation and impose additional per capita public health costs (8), highlighting the urgency of risk management. The current WHO guidelines for rabies PEP recommend a 4-dose regimen (9), but they do not adequately address risk-specific factors such as bilateral arm vaccination or hypertensive patients, nor have they established an individualized risk stratification tool, which may lead to insufficient identification of high-risk populations. Based on a retrospective cohort (2023–2025, *n* = 806) from the Animal Injury Clinic of Ningbo Ninth Hospital, this study employed LASSO regression combined with multivariate logistic regression to develop a predictive nomogram model for adverse reactions, overcoming the high-dimensional variable processing limitations typical of single-center studies. The constructed nomogram model retains the statistical rigor of logistic regression (10) while offering clinical ease of use (11). It achieves, for the first time, individualized risk stratification for adverse reactions following rabies vaccination, overcoming the dual limitations of traditional vaccine safety assessment that often “focus on mechanism over prediction” (12) and “prioritize accuracy over translation” (13). This provides the first practical and visual risk management tool for rabies PEP.

## Materials and methods

### Study population

#### Study subjects

**Figure 1:**
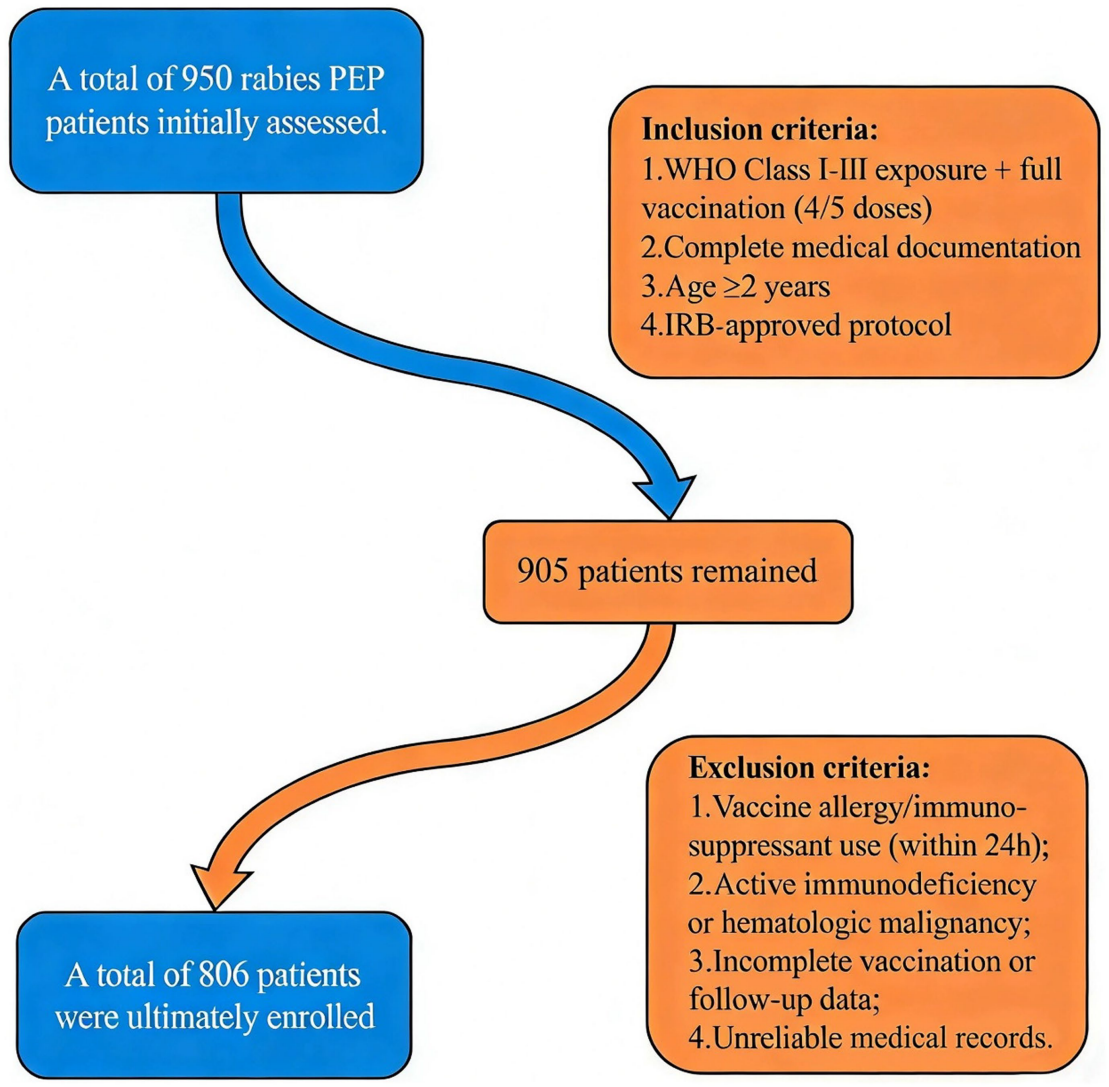
Study flowchart of patient selection criteria.

A retrospective cohort study was conducted, selecting 806 patients who received rabies post-exposure prophylaxis at the Rabies Exposure Clinic of Ningbo Ninth Hospital between September 2023 and August 2025 as the study subjects (Figure 1).

#### Inclusion criteria

1. Confirmed exposure and full vaccination: Mammalian bite/scratch with WHO-defined rabies exposure (Class I-III) and completed vaccination course (4 or 5 doses).
2. Complete medical records: Documented vaccination details, adverse reaction logs, and baseline demographics/clinical data.
3. Age ≥2 years: Covers youngest clinical cohort while ensuring immune response relevance.
4. Ethical compliance: IRB-approved retrospective design with waived consent (de-identified data).

#### Exclusion criteria

1. Contraindications: Known allergy to rabies vaccine or use of immunosuppressants within 24 h prior to vaccination.
2. Confounding medical conditions: Active immunodeficiency (e.g., HIV/AIDS), hematologic malignancies, or concurrent participation in other vaccine trials.
3. Incomplete data: Failure to complete the vaccination series or absence of follow-up records for adverse reactions beyond critical timepoints (e.g., 72 h post-vaccination).
4. Unreliable records: Missing or inconsistent documentation of vaccination details or adverse event logs.

### Ethics

This retrospective cohort study utilized de-identified data from patients vaccinated at the Rabies Exposure Clinic of Ningbo Ninth Hospital between September 2023 and August 2025. The study protocol received approval from the hospital’s Institutional Review Board with a waiver of informed consent and was conducted in accordance with the Declaration of Helsinki and Chinese ethical guidelines for biomedical research. Data security measures included restricted access through two-factor authentication and analysis on secure internal servers. To ensure data accuracy, two researchers independently performed data entry and verification, with any discrepancies resolved by a senior physician committee.

### Clinical data

A comprehensive set of multidimensional data was collected from 806 patients who presented to the Rabies Exposure Clinic of Ningbo Ninth Hospital. The data included the following categories:

#### Basic demographic characteristics

Age, sex, height, body weight, body mass index (BMI) (calculated as weight in kilograms divided by height in meters squared), and history of other vaccinations.

#### Exposure and injury profiles

Type of animal involved (domestic or stray), WHO exposure classification (Level I/II/III) (categorized per WHO guidelines: Level I, touching/feeding; Level II, minor scratches without bleeding; Level III, transdermal bites/mucous membrane exposure), exposure category (non-Level III vs. Level III), and risk classification of the exposure site (high, medium, or low).

#### Vaccination procedure details

Time interval from exposure to vaccination (hours), wound management timing (≤2 h, 2–24 h, >24 h) (defined as the time from animal exposure to the completion of standardized wound care, which included irrigation with soapy water or saline for at least 15 min, application of povidone-iodine, and assessment by a trained nurse or physician who documented the procedure in the electronic medical record), post-reconstitution vaccine storage time, baseline body temperature prior to vaccination, skinfold thickness at injection site (mm) (measured at the deltoid region using a skinfold caliper), ambient temperature and humidity during vaccination, vaccination regimen (2-dose, 4-dose, or 5-dose), injection site (left/right/bilateral deltoid) (bilateral defined as simultaneous administration in both arms), and season of vaccination.

#### Health background information

Medical history (including hypertension, diabetes, malignancies, rheumatic and autoimmune diseases, among others) and allergy history (self-reported or medically documented history of hypersensitivity to any allergen).

#### Adverse reaction monitoring

##### Type

Local reactions (redness, induration, pain) and systemic reactions (fever defined as axillary temperature ≥37.5 °C, headache, fatigue).

##### Severity grading

Graded from 1 (mild) to 5 (death) according to the U.S. FDA Toxicity Grading Scale for Healthy Adults and Adolescent Vaccine Recipients (14). Specific criteria included:

- Grade 1 (mild): Transient discomfort; no interference with normal activities.
- Grade 2 (moderate): Mild to moderate limitation in activity; minimal medical intervention required.
- Grade 3 (severe): Significant impairment; unable to perform normal activities; medical attention required.
- Grades 4–5 (life-threatening/death): As defined by the FDA scale.

##### Temporal characteristics

Time of onset (hours post-vaccination) and duration (≤24 h, 24–48 h, >48 h).

##### Surveillance method and detection window

Adverse events were monitored through a combined passive and active surveillance system. Passive surveillance was conducted by instructing all patients or their guardians to report any adverse symptoms occurring at any time after vaccination during the follow-up period. Additionally, active telephone follow-ups were initiated for all patients at 24 h and 72 h post-vaccination to systematically solicit information on predefined adverse events. This defined the primary detection window for adverse events as 0 to 72 h following each vaccine dose.

##### Management and outcome

Management measures (none, cold compress, medication) and final outcome (resolved or persistent).

### Statistical analysis

The overall analytical strategy proceeded in the following sequence: (1) initial univariate screening of candidate variables; (2) variable selection using LASSO regression with 10-fold cross-validation; (3) construction of a multivariable logistic regression model with the selected variables; (4) development and presentation of a clinical nomogram based on the final model; (5) internal validation via bootstrap resampling; and (6) performance evaluation using discrimination, calibration, and decision curve analyses. This prespecified sequence was adhered to prior to model fitting to ensure methodological rigor and reproducibility.

All analyses were conducted using SPSS (version 22.0) and R software (version 4.2.0). The dataset used for this analysis was a complete-case dataset with no missing values for any of the variables of interest; therefore, no imputation or deletion procedures for handling missing data were necessary. No specific outlier treatment was applied as all continuous variable values fell within clinically plausible ranges upon initial data screening. Continuous variables that did not follow a normal distribution are presented as median and interquartile range (IQR), and comparisons between groups were conducted using the Mann–Whitney *U* test. Categorical variables are summarized as frequencies and percentages, and group differences were assessed using the chi-square test. The dataset was randomly split into training and testing sets at a ratio of 7:3. Variable selection was performed using least absolute shrinkage and selection operator (LASSO) regression. To assess potential multicollinearity among the predictor variables, the variance inflation factor (VIF) was calculated for each variable in the multivariable logistic regression model. A VIF value of less than 5 was considered to indicate no significant multicollinearity. Multivariable logistic regression was then applied to identify independent predictors. Based on the resulting model, a nomogram was developed using the “rms” package in R to facilitate clinical prediction. The discriminative ability of the model was evaluated using receiver operating characteristic (ROC) curve analysis. Calibration was assessed via calibration curves. Decision curve analysis (DCA) was employed to quantify net benefit across a range of threshold probabilities, thereby identifying the optimal intervention threshold. Subgroup analyses were performed to assess the consistency of the association between high-risk exposure (Grade III) and adverse reaction risk. Within each subgroup, univariable logistic regression was used to estimate odds ratios (ORs) and 95% confidence intervals (CIs). Effect modification was formally tested by including a multiplicative interaction term in the multivariable model, with a two-sided *p*-value for interaction <0.10 considered statistically significant. A two-sided *p*-value <0.05 was considered statistically significant.

## Results

The dataset was randomly partitioned into a training set (70%) and an independent testing set (30%) for model development and validation, respectively. Comparative analysis revealed no statistically significant differences in baseline demographic, clinical, or exposure-related characteristics between the training and testing cohorts (all *p* > 0.05; Table 1), confirming adequate balance and comparability between the two groups. Key variables assessed included age, gender, BMI, wound management timing, exposure level, vaccination details, and medical history. The consistency in variable distribution across sets supports the robustness of the subsequent modeling process and the generalizability of the derived predictions.

**Table 1:** Comparison of baseline characteristics between training and testing sets.

Characteristic	Total cohort (*n* = 806)	Training set (*n* = 564)	Testing set (*n* = 242)	*χ*^2^/z-value	*p*-value
Gender, *n* (%)				0.004	0.950
Male	385 (47.77)	269 (47.7)	116 (47.93)		
Female	421 (52.23)	295 (52.3)	126 (52.07)		
Age (years)	34 (23,48)	34 (23,47)	33.5 (23,51)	−0.481	0.631
BMI (kg/m^2^)	22.7 (21.1,24.8)	22.7 (21.2,24.8)	22.9 (21.1,25)	−0.563	0.573
Prior non-rabies vaccination, *n* (%)				-	-
No	806 (100)	564 (100)	242 (100)		
Yes	0 (0)	0 (0)	0 (0)		
Time to vaccination (h)	3.2 (2.4,4)	3.2 (2.4,4.0)	3.1 (2.3,3.9)	−1.322	0.186
Wound management timing, *n* (%)				0.606	0.738
≤2 h	142 (17.62)	103 (18.26)	14 (20.29)		
2–24 h	646 (80.15)	448 (79.43)	53 (76.81)		
>24 h	18 (2.23)	13 (2.30)	2 (2.90)		
Animal type, *n* (%)				0.310	0.577
Stray	636 (78.91)	448 (79.43)	188 (77.69)		
Domestic	170 (21.09)	116 (20.57)	54 (22.31)		
WHO exposure grade, *n* (%)				0.096	0.757
Non-III	602 (74.69)	423 (75)	179 (73.97)		
III	204 (25.31)	141 (25)	63 (26.03)		
Exposure site risk, *n* (%)				2.741	0.254
Low	321 (39.83)	235 (41.67)	86 (35.54)		
Medium	340 (42.18)	232 (41.13)	108 (44.63)		
High	145 (17.99)	97 (17.20)	48 (19.83)		
Season of vaccination, *n* (%)				3.214	0.360
Spring	200 (24.81)	132 (23.40)	68 (28.10)		
Summer	415 (51.49)	300 (53.19)	115 (47.52)		
Autumn	92 (11.41)	66 (11.70)	26 (10.74)		
Winter	99 (12.28)	66 (11.70)	33 (13.64)		
Post-reconstitution storage time (h)	0 (0,0)	0 (0,0)	0 (0,0)	0.000	1.000
Baseline body temperature (°C)	36.5 (36.4,36.7)	36.5 (36.4,36.7)	36.5 (36.4,36.7)	−0.511	0.609
Skinfold thickness (mm)	25 (20,35)	25 (20,35)	25 (20,35)	−1.907	0.056
Ambient temperature (°C)	22 (21,23)	22 (21,23)	22 (20,23)	−0.656	0.512
Ambient humidity (%)	56 (51,61)	56 (51.5,61)	56 (51,61)	−0.208	0.835
Vaccination regimen, *n* (%)				0.482	0.786
2-dose	364 (45.16)	258 (45.74)	33 (47.83)		
4-dose	402 (49.88)	277 (49.11)	32 (46.38)		
5-dose	40 (4.96)	29 (5.14)	4 (5.8)		
Injection site, *n* (%)				0.106	0.948
Left deltoid	330 (40.94)	233 (41.31)	97 (40.08)		
Right deltoid	56 (6.95)	39 (6.91)	17 (7.02)		
Bilateral deltoid	420 (52.11)	292 (51.77)	128 (52.89)		
Comorbidities					
Hypertension, *n* (%)	63 (7.82)	45 (7.98)	18 (7.44)	0.069	0.793
Diabetes, *n* (%)	17 (2.11)	10 (1.77)	7 (2.89)	1.028	0.311
Malignancy, *n* (%)	31 (3.85)	21 (3.72)	10 (4.13)	0.077	0.782
Rheumatic/autoimmune, *n* (%)	21 (2.61)	16 (2.84)	5 (2.07)	0.396	0.529
Allergy history				0.526	0.468
No allergy, *n* (%)	760 (94.29)	534 (94.68)	60 (86.96)		
Allergy reported, *n* (%)	46 (5.71)	30 (5.32)	9 (13.04)		

Comparative analysis revealed significant differences between patients with and without adverse effects in several key exposure and clinical variables (Table 2). Specifically, shorter time to vaccination, delayed wound management, stray animal exposure, WHO Grade III injuries, reduced skinfold thickness at the injection site, bilateral deltoid vaccination, and a positive allergy history were significantly associated with higher adverse event rates (*p* < 0.05). In contrast, demographic factors such as age, sex, and BMI showed no statistically significant associations.

**Table 2:** Comparison of baseline characteristics between patients with and without adverse effects in the training cohort.

Characteristic	No adverse effects (*n* = 426)	Adverse effects (*n* = 138)	*χ*^2^/*z*-value	*p*-value
Gender, *n* (%)			0.893	0.345
Male	208 (48.83)	61 (44.20)		
Female	218 (51.17)	77 (55.80)		
Age (years)	35 (23,47)	33 (22,47)	−0.711	0.477
BMI (kg/m^2^)	22.7 (21.2,24.9)	22.65 (21.3,24.7)	−0.629	0.529
Prior non-rabies vaccination, *n* (%)			-	-
No	426 (100)	138 (100)		
Yes	0 (0)	0 (0)		
Time to vaccination (h)	3.2 (2.4,4)	3.25 (2.5,4.8)	−2.136	0.033
Wound management timing, *n* (%)			16.750	< 0.001
≤2 h	89 (20.89)	14 (10.14)		
2–24 h	332 (77.93)	116 (84.06)		
>24 h	5 (1.17)	8 (5.80)		
Animal type, *n* (%)			5.170	0.023
Stray	329 (77.23)	119 (86.23)		
Domestic	97 (22.77)	19 (13.77)		
WHO exposure grade, *n* (%)			19.456	< 0.001
Non-III	339 (79.58)	84 (60.87)		
III	87 (20.42)	54 (39.13)		
Exposure site risk, *n* (%)			1.204	0.548
Low	183 (42.96)	52 (37.68)		
Medium	171 (40.14)	61 (44.2)		
High	72 (16.9)	25 (18.12)		
Season of vaccination, *n* (%)			1.471	0.689
Spring	104 (24.41)	28 (20.29)		
Summer	225 (52.82)	75 (54.35)		
Autumn	47 (11.03)	19 (13.77)		
Winter	50 (11.74)	16 (11.59)		
Post-reconstitution storage time (h)	0 (0,0)	0 (0,0)	0.000	1.000
Baseline body temperature (°C)	36.5 (36.4,36.7)	36.5 (36.4,36.7)	−1.056	0.291
Skinfold thickness (mm)	30 (25,35)	20 (15,25)	−9.428	< 0.001
Ambient temperature (°C)	22 (21,24)	22 (20,23)	−0.213	0.831
Ambient humidity (%)	56 (51,62)	57 (52,61)	−0.299	0.765
Vaccination regimen, *n* (%)			6.666	0.036
2-dose	182 (42.72)	76 (55.07)		
4-dose	222 (52.11)	55 (39.86)		
5-dose	22 (5.16)	7 (5.07)		
Injection site, *n* (%)			7.116	0.028
Left deltoid	187 (43.9)	46 (33.33)		
Right deltoid	32 (7.51)	7 (5.07)		
Bilateral deltoid	207 (48.59)	85 (61.59)		
Comorbidities				
Hypertension, *n* (%)	29 (6.81)	16 (11.59)	3.253	0.071
Diabetes, *n* (%)	9 (2.11)	1 (0.72)	0.494	0.482
Malignancy, *n* (%)	16 (3.76)	5 (3.62)	0.005	0.943
Rheumatic/autoimmune, *n* (%)	10 (2.35)	6 (4.35)	0.875	0.350
Allergy history			11.176	0.001
No allergy, *n* (%)	411 (96.48)	123 (89.13)		
Allergy reported, *n* (%)	15 (3.52)	15 (10.87)		

**Figure 2:**
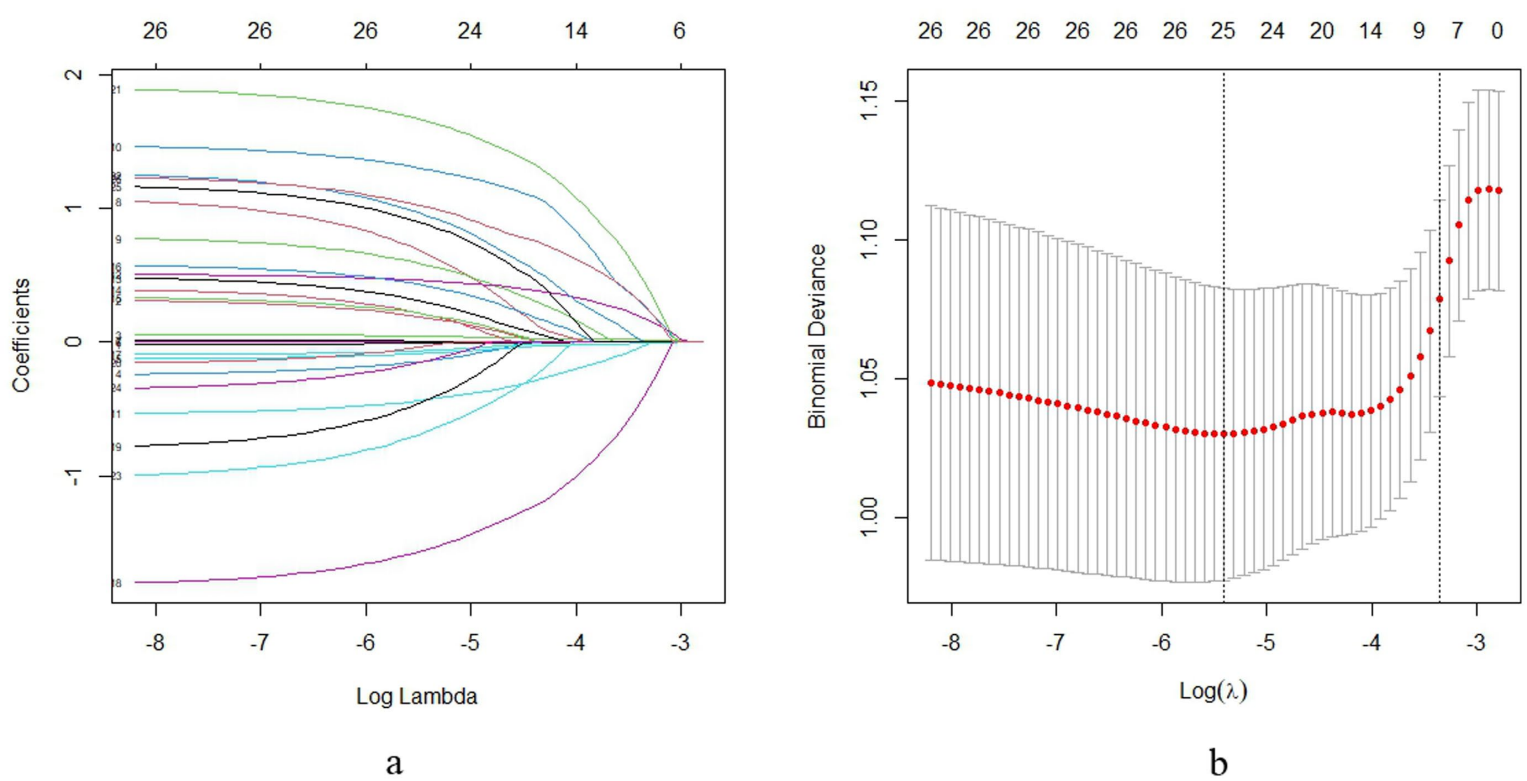
LASSO regression for feature selection and cross-validation [**(a)** coefficient paths; **(b)** binomial deviance].

The predictive model development employed LASSO (Least Absolute Shrinkary and Selection Operator) regression to systematically identify key predictors of adverse reactions following rabies vaccination from an initial set of 16 candidate variables. LASSO regression was implemented using the glmnetpackage (version 4.1–8) in R software (version 4.2.0). The model was fitted with the family parameter set to “binomial” for binary outcomes, and the regularization path was computed over a sequence of 100 *λ* values (nlambda = 100) with the elastic net mixing parameter *α* fixed at 1 (pure LASSO penalty). Through 10-fold cross-validation using the lambda.1se criterion, the analysis determined an optimal regularization parameter [*λ* = 0.035, corresponding to log(λ) ≈ −3.4] that balanced model parsimony with predictive accuracy, ultimately selecting eight clinically relevant predictors with non-zero coefficients. The lambda.1se criterion selects the largest *λ* value within one standard error of the minimum binomial deviance, promoting a sparser and more stable model. The coefficient path analysis (Figure 2a) demonstrated systematic adjustment of variable contributions as the penalty parameter increased, with final selection occurring at the predetermined *λ* threshold that optimized model performance. The corresponding cross-validation curve (Figure 2b) confirmed the optimal λ value selection through minimum binomial deviance criteria. These predictors included the time interval from exposure to vaccination initiation, wound management timing, type of exposure animal (stray versus domestic), WHO exposure grade classification, skinfold thickness at the injection site, vaccination regimen (2, 4, or 5 doses), site of vaccination (unilateral or bilateral administration), and history of allergic reactions. This analytical approach effectively reduced model complexity while maintaining predictive power by eliminating less informative variables and retaining those with both statistical significance and clinical relevance, thereby establishing a robust foundation for clinical risk assessment and decision-making regarding rabies vaccination protocols. The selected variables collectively represent exposure characteristics, vaccination parameters, and individual patient factors that influence adverse reaction likelihood.

The LASSO coefficient paths (Figure 2a) illustrate how variable coefficients progressively shrink toward zero as regularization intensity increases (log *λ* range: −8 to −3). The vertical numeric labels (26 to 0) indicate the number of retained variables at each *λ* value. Figure 2b displays the corresponding binomial deviance curve, where the optimal *λ* (log *λ* ≈ −5) was selected at the minimum deviance point with one standard error constraint. This selection retained exactly eight non-zero coefficients, corresponding to clinically relevant predictors identified in univariate analysis. The U-shaped deviance curve confirms appropriate model tuning, balancing predictive accuracy with generalizability by avoiding both overfitting and excessive sparsity.

Multivariable logistic regression analysis identified six independent predictors of adverse reactions (Table 3). The full model included eight candidate variables; two variables (time from exposure to vaccination and type of exposure animal) did not show a statistically significant independent association (*p* > 0.05) in the final model and are not displayed in Table 3. Bilateral deltoid administration (OR = 4.926, 95%CI: 2.367–10.252, *p* < 0.001) and allergy history (OR = 5.065, 95%CI:1.992–12.874, *p* = 0.001) emerged as the strongest risk factors. The four-dose regimen demonstrated significant protection compared to the two-dose regimen (OR = 0.16, 95%CI:0.08–0.33, *p* < 0.001). Other significant predictors included delayed wound management (OR = 2.131, 95%CI: 1.082–4.197, *p* = 0.029), higher WHO exposure grades (OR = 1.368, 95%CI: 1.048–1.785, *p* = 0.021), and reduced skinfold thickness at injection sites (OR = 0.885, 95%CI: 0.857–0.913, *p* < 0.001). Time from exposure to vaccination, animal type, and unilateral right deltoid administration showed no significant associations in the final model.

**Table 3:** Multivariable logistic regression analysis of factors associated with adverse reactions to rabies vaccination.

Variable	B	S.E.	Wald	*p*-value	OR	95%CI	
						Lower Limit (LL)	Upper Limit (UL)
Exposure grade	0.313	0.136	5.331	0.021	1.368	1.048	1.785
Vaccination site							
Left deltoid					1.000		
Right deltoid	−0.010	0.527	0.001	0.985	0.99	0.352	2.785
Bilateral deltoid	1.595	0.374	18.186	<0.001	4.926	2.367	10.252
Allergy history	1.622	0.476	11.616	0.001	5.065	1.992	12.874
Wound management timing	0.757	0.346	4.788	0.029	2.131	1.082	4.197
Vaccination regimen							
2-dose					1.000		
4-dose	−1.829	0.373	24.122	<0.001	0.160	0.077	0.333
5-dose	−0.651	0.560	1.351	0.245	0.522	0.174	1.563
Skinfold thickness (mm)	−0.122	0.016	57.814	<0.001	0.885	0.857	0.913
Time to vaccination (h)	0.023	0.025	0.848	0.357	1.024	0.974	1.075
Animal type	−0.439	0.316	1.927	0.165	0.645	0.347	1.198
Constant	0.364	0.854	0.182	0.670	1.439		

The model was constructed following variable selection via least absolute shrinkage and selection operator (LASSO) regression. The full model included the following candidate variables: time from exposure to vaccination, wound management timing, type of exposure animal (stray vs. domestic), WHO exposure grade, skinfold thickness at injection site, vaccination regimen, vaccination site (unilateral/bilateral), and allergy history. Only variables with a statistically significant association (*p* < 0.05) in the final multivariable model are displayed in this table.

**Figure 3:**
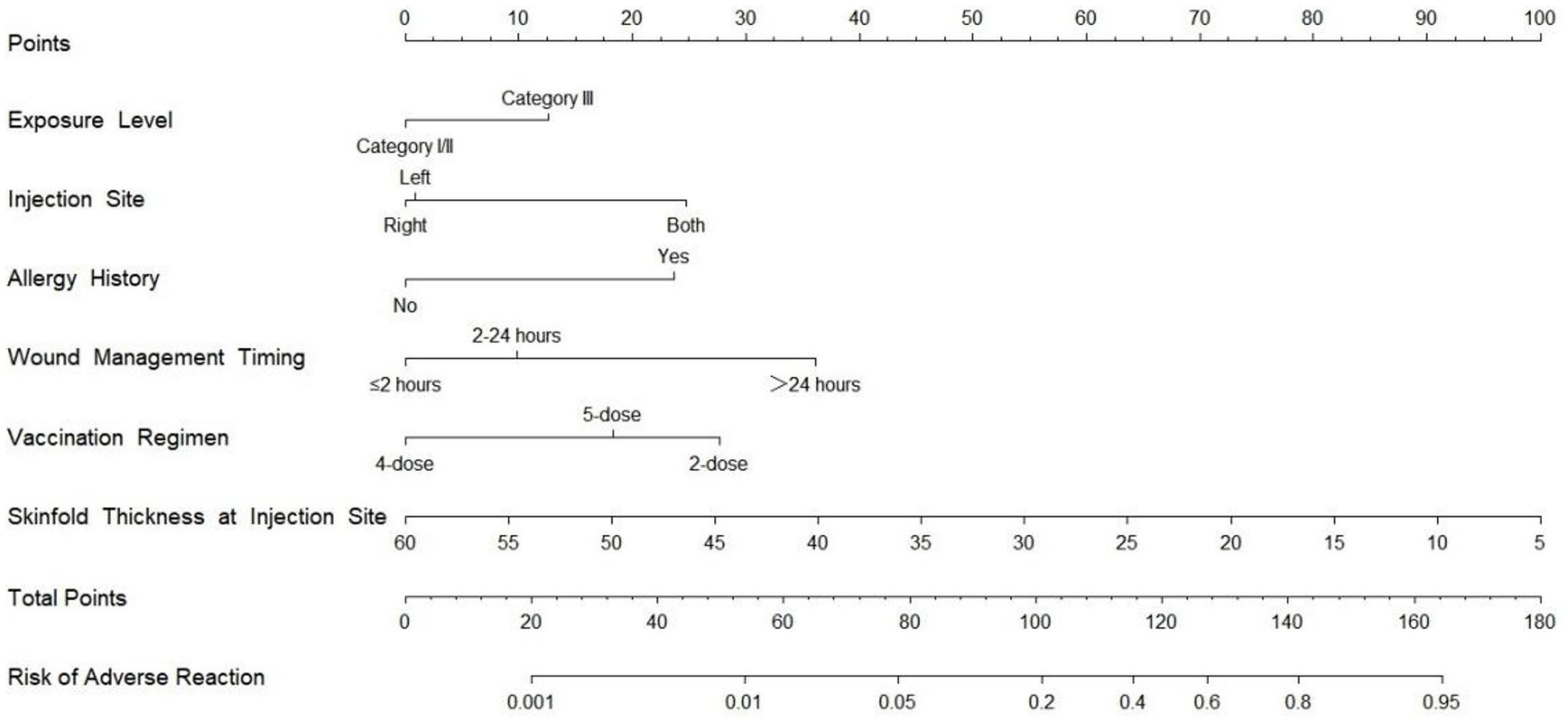
Nomogram for predicting adverse reaction risk following rabies vaccination.

The nomogram was developed based on six independent predictors identified through LASSO-logistic regression analysis (Figure 3). It incorporates exposure level (Category I/II/III), injection site (left/right/bilateral deltoid), allergy history (yes/no), wound management timing (≤2 h/2–24 h/>24 h), vaccination regimen (2-dose/4-dose/5-dose), and skinfold thickness at injection site (10–60 mm). To use the nomogram, the corresponding points for each variable are summed to obtain a total score, which is then projected to the risk axis at the bottom to determine the individual probability of adverse reactions, ranging from 0.1 to 95%. The nomogram thus provides a direct visual translation of the multivariate logistic regression model, where the length of each variable’s scale is proportional to its contribution to the outcome. The total points scale effectively represents the linear predictor of the model. The primary clinical value of this tool lies in its interpretability: the calculated probability quantifies an individual’s baseline risk prior to vaccination. This risk estimate can directly inform clinical decisions, such as intensifying monitoring for high-risk patients (e.g., those with a predicted probability >20%), opting for preventive strategies like unilateral deltoid injection, or reassuring low-risk individuals. This transforms the model from a statistical abstraction into a practical decision-support tool at the point of care.

**Figure 4:**
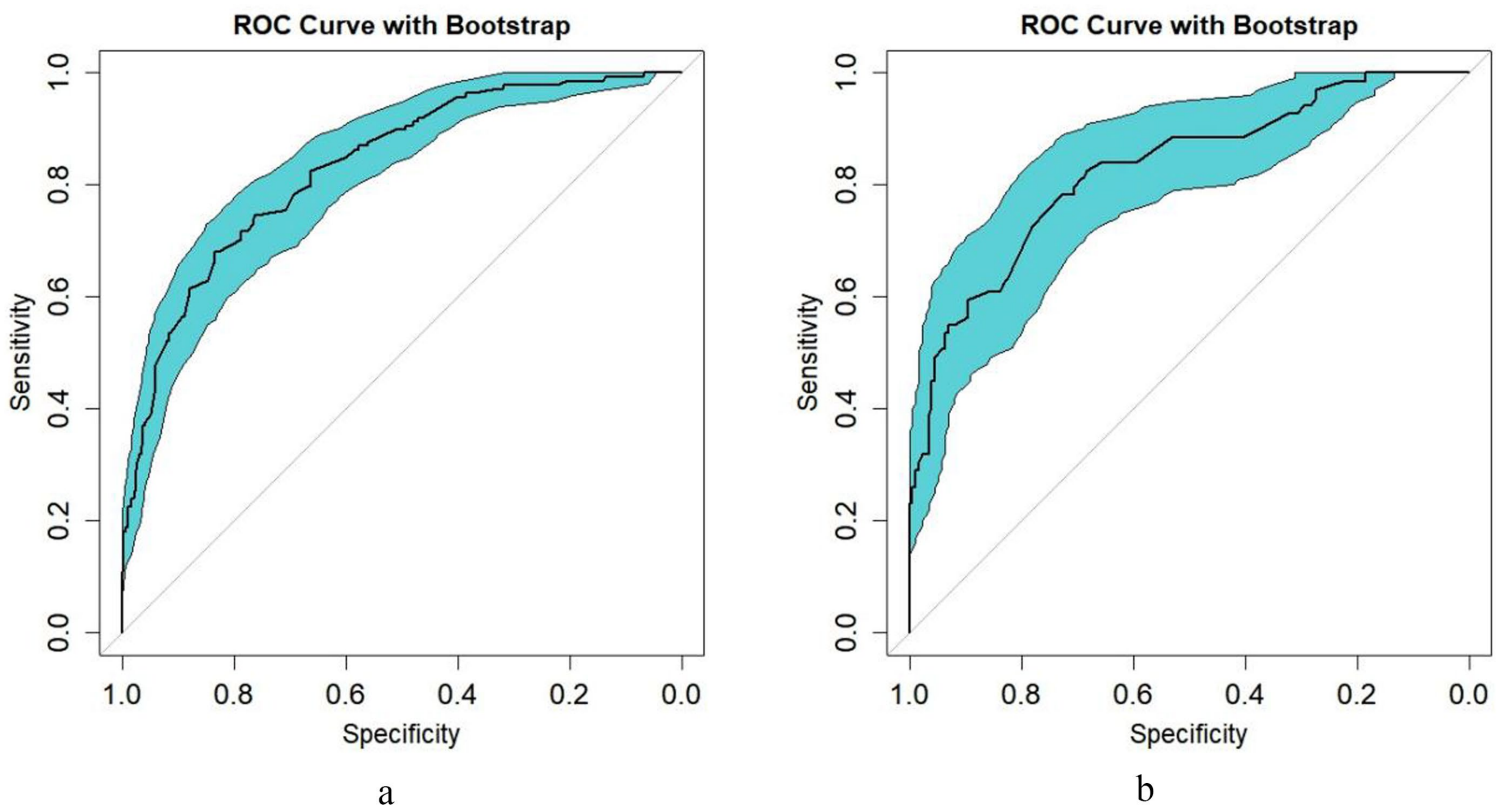
Bootstrap-validated ROC curve analysis of the predictive model [**(a)** training set; **(b)** test set].

The predictive performance of the nomogram was evaluated using receiver operating characteristic (ROC) analysis in both training (Figure 4a) and testing (Figure 4b) cohorts. In the training set, the model demonstrated a discriminative ability with an area under the curve (AUC) of 0.826 (95% CI: 0.786–0.866). The testing set validation showed comparable performance, achieving an AUC of 0.817 (95% CI: 0.756–0.878). Both ROC curves were accompanied by bootstrap confidence intervals (light blue shading), indicating stable estimation of model performance. The diagonal dashed line represents the reference for a non-informative model (AUC = 0.5). The close agreement between training and testing AUC values confirms the model’s robust predictive capability and generalizability across different patient populations.

**Figure 5:**
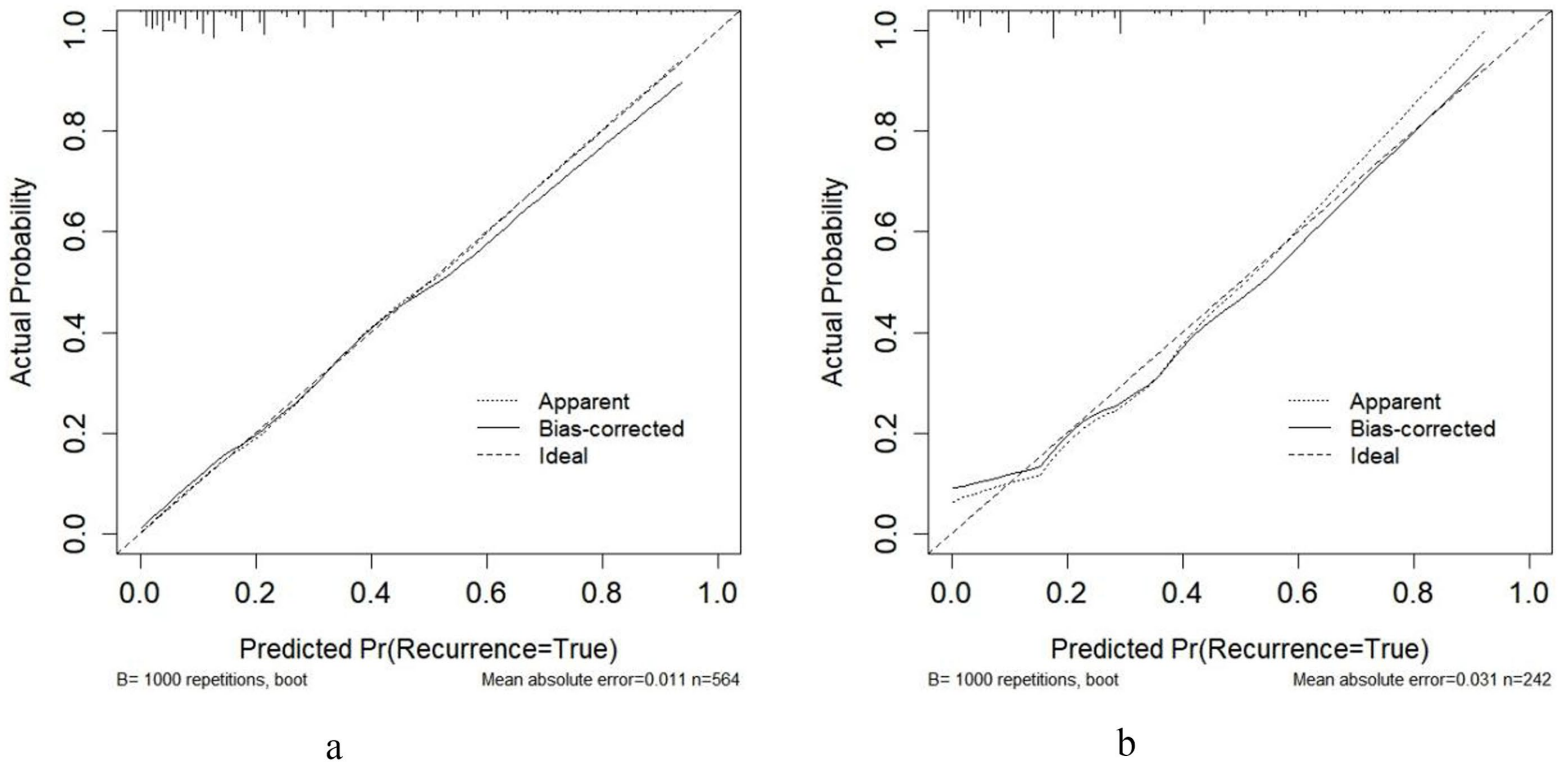
Bootstrap-validated calibration curve analysis of the predictive model [**(a)** training set; **(b)** test set].

**Figure 6:**
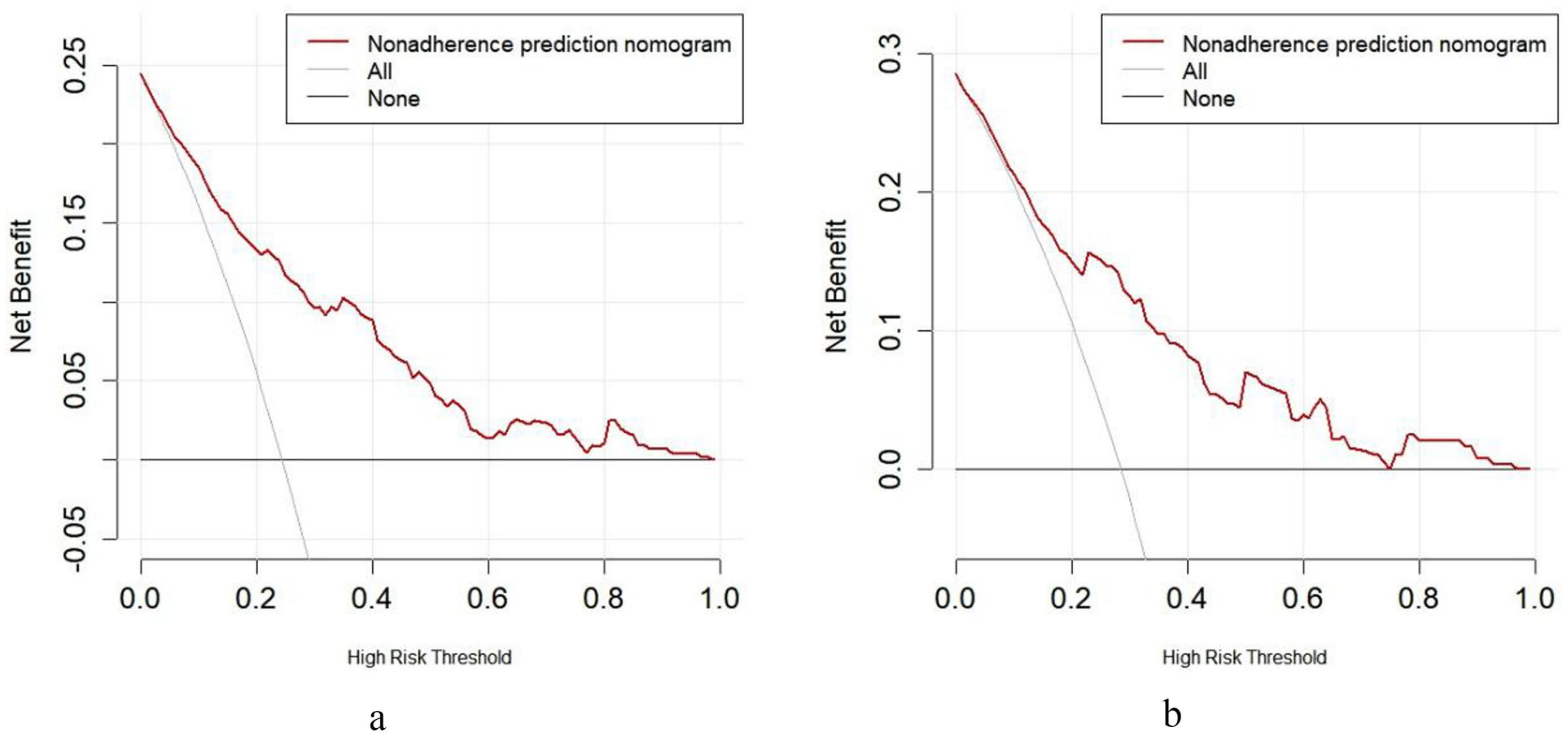
Bootstrap-validated decision curve analysis of the predictive model [**(a)** training set; **(b)** test set].

The calibration curves demonstrate the agreement between predicted probabilities and observed outcomes of adverse reactions following rabies vaccination. In the training set (Figure 5a), the bias-corrected curve closely followed the ideal reference line, with a mean absolute error of 0.011 (*n* = 564, bootstrap repetitions = 1,000). The testing set (Figure 5b) showed good calibration consistency with a mean absolute error of 0.031 (*n* = 242, bootstrap repetitions = 1,000), though minor deviations were observed at lower probability ranges. Hosmer-Lemeshow goodness-of-fit tests further validated the calibration accuracy, with training set results (*χ*^2^ = 3.097, *p* = 0.928) and testing set results (*χ*^2^ = 11.727, *p* = 0.164) both confirming excellent model fit. These comprehensive calibration assessments indicate that the nomogram provides well-calibrated risk predictions across different patient populations, confirming its reliability in translating predicted probabilities to actual clinical outcomes.
Decision curve analysis was performed to evaluate the clinical utility of the nomogram model across different risk thresholds. In the training set (Figure 6a), the nomogram demonstrated superior net benefit compared to “All” and “None” strategies within a high-risk threshold range of approximately 0.04–0.97. The testing set (Figure 6b) showed consistent performance, with the nomogram maintaining higher net benefit than both reference strategies across thresholds ranging from 0.0 to 0.9. In both cohorts, the nomogram’s net benefit gradually decreased as the risk threshold increased but remained above the reference curves throughout most intervals. These results indicate that the nomogram provides positive net benefits over a wide range of risk thresholds in both training and testing populations, supporting its clinical utility for decision-making regarding rabies vaccination adverse reaction risk assessment.

**Figure 7:**
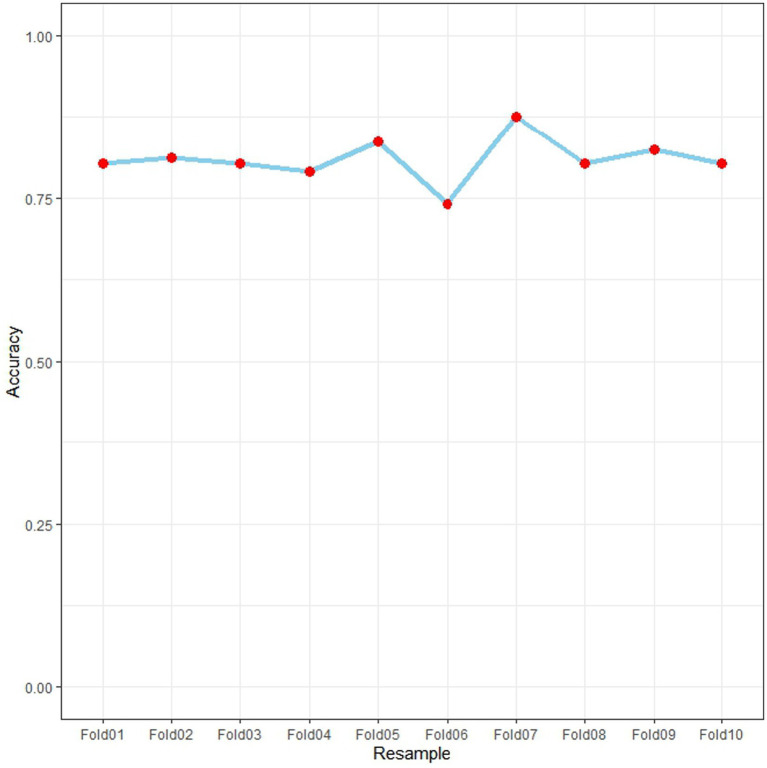
Model accuracy variation across 10-fold cross-validation.

The nomogram model underwent ten-fold cross-validation to assess its predictive performance and generalizability across different data subsets (Figure 7). This process involved randomly partitioning the entire dataset into ten equally sized folds. Sequentially, each fold was used once as the validation set, while the remaining nine folds constituted the training set for model building and performance calculation. The final performance metrics represent the average across all ten iterations. The model achieved an overall accuracy of 0.800, indicating correct classification of 80.0% of samples on average. Accuracy values across the ten folds (Fold01-Fold10) ranged from 0.75 to 0.85, demonstrating consistent performance with minimal fluctuation around the mean value. The stability observed across all resamples, with no significant performance variations between batches, confirms the model’s robustness without evidence of overfitting or underfitting. These results support the reliability of the nomogram for clinical prediction applications.

Stratified analysis was conducted to evaluate the association between high-risk exposure (Grade III) and adverse reaction risk across various patient subgroups (Table 4). The results demonstrated that high-risk exposure significantly increased adverse reaction risk (OR >1, *p* < 0.05) in most subgroups. Several factors showed particularly strong associations: hypertension history (OR = 4.429, 95%CI: 1.381–14.206), autumn vaccination (OR = 3.741, 95%CI: 1.374–10.183), bilateral deltoid vaccination (OR = 2.412, 95%CI: 1.534–3.794), and ambient temperature <22 °C (OR = 2.426, 95%CI: 1.382–4.258).

**Table 4:** Stratified analysis of the association between high-risk exposure (Grade III) and adverse reaction risk across different subgroups.

Variable category	Subgroup variable	B	S.E.	Wald	*p*	OR	95%CI	
							Lower limit (LL)	Upper limit (UL)
A. Demographic factors								
	Gender, *n* (%)							
	Male	0.930	0.263	12.538	<0.001	2.535	1.515	4.242
	Female	0.608	0.237	6.573	0.010	1.837	1.154	2.925
	Age (years)							
	<34	0.684	0.248	7.579	0.006	1.981	1.218	3.223
	≥34	0.832	0.249	11.128	0.001	2.298	1.41	3.748
	BMI (kg/m^2^)							
	<22.7	0.567	0.312	3.297	0.069	1.762	0.956	3.249
	≥22.7	0.848	0.213	15.77	<0.001	2.334	1.536	3.546
B. Vaccination parameters								
	Time to vaccination (h)							
	<3.2	0.633	0.254	6.215	0.013	1.883	1.145	3.097
	≥3.2	0.874	0.245	12.768	<0.001	2.398	1.484	3.873
	Wound management timing (h)							
	≤2	0.206	0.472	0.191	0.662	1.229	0.488	3.096
	2–24	0.996	0.194	26.274	<0.001	2.707	1.85	3.962
	>24	−0.875	1.076	0.662	0.416	0.417	0.051	3.435
	Animal type, *n* (%)							
	Stray	0.807	0.193	17.467	<0.001	2.241	1.535	3.271
	Domestic	0.424	0.446	0.903	0.342	1.528	0.637	3.662
	Exposure site risk, *n* (%)							
	Low	0.861	0.293	8.623	0.003	2.366	1.332	4.203
	Medium	0.802	0.263	9.303	0.002	2.23	1.332	3.733
	High	0.444	0.409	1.179	0.278	1.559	0.699	3.477
C. Vaccination details								
	Season of vaccination, *n* (%)							
	Spring	0.512	0.377	1.842	0.175	1.668	0.797	3.492
	Summer	0.749	0.241	9.656	0.002	2.114	1.319	3.391
	Autumn	1.319	0.511	6.669	0.010	3.741	1.374	10.183
	Winter	0.744	0.527	1.996	0.158	2.105	0.75	5.913
D. Clinical and environmental factors								
	Baseline body temperature (°C)							
	<36.5	0.756	0.265	8.134	0.004	2.129	1.267	3.579
	≥36.5	0.764	0.236	10.499	0.001	2.148	1.353	3.41
	Skinfold thickness (mm)							
	<25	0.688	0.286	5.796	0.016	1.989	1.136	3.481
	≥25	0.728	0.232	9.855	0.002	2.071	1.314	3.262
	Ambient temperature (°C)							
	<22	0.886	0.287	9.534	0.002	2.426	1.382	4.258
	≥22	0.676	0.223	9.152	0.002	1.966	1.269	3.047
	Ambient humidity (%)							
	<56	0.644	0.273	5.555	0.018	1.904	1.115	3.254
	≥56	0.859	0.232	13.735	<0.001	2.362	1.499	3.721
	Vaccination regimen, *n* (%)							
	2-dose	0.649	0.254	6.531	0.011	1.913	1.163	3.146
	4-dose	0.913	0.258	12.482	<0.001	2.491	1.501	4.134
	5-dose	0.588	0.837	0.494	0.482	1.800	0.349	9.278
	Injection site, *n* (%)							
	Left deltoid	0.569	0.309	3.385	0.066	1.766	0.964	3.236
	Right deltoid	0.426	0.669	0.405	0.525	1.531	0.412	5.68
	Bilateral deltoid	0.881	0.231	14.532	<0.001	2.412	1.534	3.794
E. Comorbidities and history								
	Hypertension history							
	No	0.676	0.187	13.088	<0.001	1.967	1.363	2.837
	Yes	1.488	0.595	6.261	0.012	4.429	1.381	14.206
	Diabetes history							
	No	0.759	0.178	18.138	<0.001	2.136	1.506	3.028
	Yes	0.811	1.167	0.483	0.487	2.25	0.229	22.144
	Malignancy history							
	No	0.737	0.178	17.073	<0.001	2.091	1.474	2.966
	Yes	1.482	1.116	1.763	0.184	4.40	0.494	39.21
	Rheumatic/autoimmune history							
	No	0.725	0.18	16.208	<0.001	2.064	1.45	2.937
	Yes	1.322	0.946	1.953	0.162	3.750	0.587	23.936
	Allergy history							
	No	0.952	0.182	27.453	<0.001	2.59	1.814	3.697
	Yes	−0.442	0.829	0.284	0.594	0.643	0.127	3.261

Conversely, no significant association was observed in specific subgroups, including patients with wound management time >24 h (OR = 0.417, 95%CI: 0.051–3.435) and those with allergy history (OR = 0.643, 95%CI: 0.127–3.261). Other notable associations included BMI ≥ 22.7 kg/m^2^ (OR = 2.334, 95%CI: 1.536–3.546), vaccination time ≥3.2 h (OR = 2.398, 95%CI: 1.484–3.873), and exposure to stray animals (OR = 2.241, 95%CI: 1.535–3.271).

The analysis covered multiple stratification variables including demographic characteristics, vaccination parameters, environmental factors, and medical history, with consistent results observed across most subgroups, confirming the robustness of the association between high-risk exposure and adverse reaction risk.

The analysis of adverse reaction profiles revealed that fever (13.4%) and local pain (8.44%) were the most common clinical manifestations among 806 vaccinated patients (Table 5). The majority of reactions were mild, with 91.35% classified as grade 1–2 in severity. The reactions demonstrated self-limiting characteristics, as 63.46% resolved within 24–48 h, and 86.54% of cases required no specific intervention. Notably, all documented adverse reaction cases achieved complete resolution without sequelae, with no reports of persistent symptoms. The median time to symptom onset was 6 h (IQR: 3–8), indicating relatively rapid development of post-vaccination reactions.

**Table 5:** Clinical characteristics and outcomes of adverse reactions following rabies vaccination.

Characteristic	*n* (%)/M (IQR)
Adverse reaction type	
Redness/swelling	5 (0.62)
Induration/hardening	1 (0.12)
Pain	68 (8.44)
Fever	108 (13.4)
Headache	1 (0.12)
Fatigue	23 (2.85)
Time to onset (h)	6 (3,8)
Severity grade	
1	77 (37.02)
2	113 (54.33)
3	16 (7.69)
4	2 (0.96)
5	0 (0)
Duration (h)	
≤24	24 (11.54)
24–48	132 (63.46)
>48	52 (25.00)
Management	
No intervention	180 (86.54)
Cold compress	12 (5.77)
Medication	16 (7.69)
Outcome	
Complete resolution	208 (100)
Persistent symptoms	0 (0)

**Figure 8:**
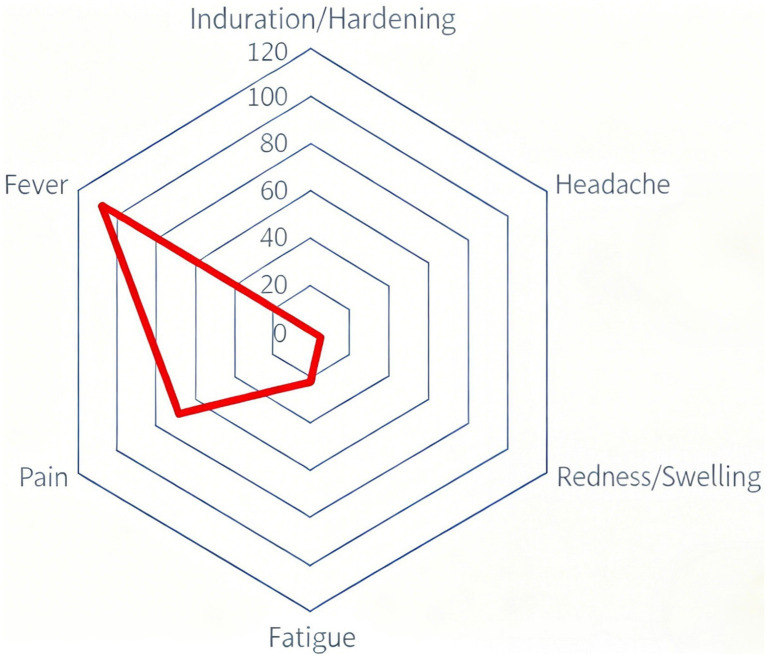
Distribution of adverse reaction types following rabies vaccination.

The radar chart visually displays the incidence distribution of six major adverse reactions, with fever demonstrating the highest incidence rate as indicated by its vertex extending furthest from the center (Figure 8). Pain shows the second highest incidence with a distinct vertex position, while induration and headache exhibit the lowest rates with vertices closest to the chart center. Redness/swelling and fatigue present intermediate incidence levels between these extremes. The radial axis scales from 0 to 120, clearly illustrating the relative frequency distribution among different reaction types. This visualization reinforces the predominant pattern of fever and pain as the most common adverse reactions following rabies vaccination.

## Discussion

This large-scale clinical investigation provides the first systematic elucidation of risk and protective factors associated with adverse reactions following rabies vaccination, a contribution that aligns with and extends the global pharmacovigilance priorities for rabies vaccines outlined by the World Health Organization (WHO) (15). It demonstrates that WHO Grade III high-risk exposure (reflecting elevated viral load and tissue damage) (16), bilateral deltoid administration (indicating expanded antigen exposure and enhanced immune stimulation) (17), positive allergy history (suggesting individual immunological hyperreactivity) (18, 19), and delayed wound management (>24 h) (20, 21) constitute independent risk predictors, while the 4-dose vaccination regimen (potentially reducing cumulative antigenic stimulation through abbreviated course) (22) and increased skinfold thickness at injection site (≥25 mm) (possibly modulating local pharmacokinetics) (23) exhibit protective effects. The developed nomogram prediction model, integrating these six clinically actionable indicators, achieves individualized risk stratification with excellent discriminative capacity (AUC 0.826 in training set) and calibration precision (mean absolute error 0.011), providing clinicians with a quantitative tool for pre-vaccination risk counseling, personalized monitoring strategies, and targeted intervention planning. This comprehensive approach bridges epidemiological findings with clinical practice by identifying both modifiable (e.g., vaccination technique, wound management timing) and non-modifiable (e.g., allergy history, anatomical characteristics) factors that collectively shape adverse reaction profiles, thereby establishing an evidence-based framework for precision medicine in rabies vaccination management and offering a foundation for future mechanistic investigations and protocol optimization.

While the observational nature of this study precludes causal inference, the robust associations identified invite mechanistic hypotheses for future testing. This study systematically identified bilateral deltoid vaccination as a significant risk factor for adverse reactions. This finding directly challenges a common clinical practice and necessitates an urgent reassessment of its risk–benefit ratio. This observation is supported by recent studies specifically focusing on rabies vaccine safety profiles, which have similarly identified procedural factors as key determinants of adverse outcomes (24, 25). WHO Grade III exposure, a well-established risk factor, is biologically plausible due to higher viral loads from severe animal injuries, leading to stronger T-cell immunity and antibody responses (26–28). The generalizability of this key risk factor is reinforced by international epidemiological data, confirming its significance across diverse populations and geographic settings (29, 30). Allergy history emerged as a key marker of individual susceptibility, reinforcing the role of atopic predisposition in modulating vaccine-related immune responses and highlighting the importance of pre-vaccination screening (31–33). Notably, delayed wound management (>24 h) was identified as a modifiable external risk factor, emphasizing the immunomodulatory role of early debridement, as delayed treatment may prolong viral antigen exposure, sustaining immune cell activation via pattern recognition receptors like Toll-like receptors and exacerbating inflammatory cascades (34–36). Collectively, these findings establish a multidimensional, clinically actionable risk assessment framework, advancing precision safety management in rabies vaccination protocols by integrating modifiable (e.g., vaccination technique, wound management timing) and non-modifiable (e.g., allergy history, exposure severity) factors into a unified predictive model with significant translational implications for clinical practice.

The 4-dose vaccination regimen and greater skinfold thickness at the injection site (≥25 mm) represent two key protective factors offering clinically actionable targets for optimizing rabies vaccination safety. The protective effect of the four-dose regimen likely stems from its shortened course, which reduces cumulative antigen exposure and physical trauma from repeated injections, thereby attenuating overall immune stress (22, 37, 38). Importantly, this simplified regimen maintains immunogenicity comparable to the conventional five-dose schedule while improving patient compliance, demonstrating enhanced safety without compromising efficacy (39, 40). The protective role of greater skinfold thickness presents a novel and clinically actionable finding. One plausible hypothesis, derived from pharmacokinetic principles, is that thicker subcutaneous adipose tissue modulates antigen absorption and distribution kinetics, which may in turn dampen the peak intensity of the systemic immune response (41–43). This hypothesis requires direct testing in future pharmacokinetic-pharmacodynamic studies. This finding implies that selecting injection sites with greater skinfold thickness, such as the thigh in lean individuals, could serve as a straightforward risk mitigation strategy. Together, these protective factors—one derived from regimen optimization and the other from anatomical characteristics—form a complementary framework for clinical intervention, emphasizing the importance of integrating vaccination protocols with individual anatomical and physiological features into a comprehensive risk assessment strategy to guide personalized safe vaccination practices.

The individualized risk prediction nomogram developed in this work represents a significant methodological advance by transcending the limitations of traditional univariate analysis, integrating four independent risk factors and two protective factors into a unified, visual risk assessment tool. Our work addresses a recognized gap in proactive vaccine safety management, contributing to the emerging field of predictive modeling for adverse events, which has been highlighted in recent methodological discussions (44, 45). Unlike conventional statistical models that provide only single *p*-values or relative risks, the nomogram translates multivariate logistic regression results into an intuitive probability scale, enabling clinicians to quantitatively calculate the risk of adverse reactions for specific patients within minutes (46, 47). A key strength of the model lies in its exceptional clinical practicality and operability; by inputting six routinely available clinical parameters—exposure grade, vaccination method, allergy history, wound management timing, vaccination regimen, and skinfold thickness—clinicians obtain a quantified risk probability (e.g., 15% or 60%), transforming the identification of high-risk patients from subjective empirical judgment to objective scientific decision-making and providing a critical basis for targeted monitoring, early warning, and preemptive interventions such as premedication with antihistamines, opting for unilateral vaccination, or enhanced observation. Furthermore, the model’s value is reinforced by its internally validated stability (via bootstrap resampling and cross-validation) and its clinically intuitive output format. It functions not merely as a statistical model but as a decision-support tool seamlessly integrated into clinical workflows, effectively bridging the gap between clinical epidemiological research and bedside practice. This study represents the first application of a nomogram model to the prediction of rabies vaccination safety risks, offering a valuable tool and direction for advancing precision medicine in this field.

Building on these key findings, it is reasonable to consider several evidence-based and clinically actionable recommendations can be proposed to systematically optimize the safety management pathway for rabies vaccination. For individuals assessed as high-risk exposure (Grade III) or those with a history of allergies, bilateral deltoid vaccination should be strictly avoided; evidence indicates that unilateral administration effectively controls the intensity and rate of antigen exposure, significantly reducing the risk of systemic inflammatory reactions, making this adjustment particularly valuable for risk mitigation in these vulnerable subgroups (48, 49). Furthermore, without compromising immunogenic efficacy, the four-dose regimen should be prioritized for specific populations—such as adolescents, individuals with allergic predispositions, or those with prior vaccine adverse reactions—as this schedule has been shown to reduce the incidence of adverse events while maintaining robust immune responses, offering a favorable balance between safety and compliance in real-world clinical settings (50, 51). Most critically, the nomogram risk prediction model developed here can be deployed as a practical decision-support tool in outpatient clinics, allowing clinicians to rapidly input exposure grade, vaccination approach, allergy history, and other variables to generate individualized risk scores, enabling real-time risk stratification and early identification of high-risk patients. Those with predicted risk probabilities exceeding 20% should receive intensified post-vaccination monitoring and potential preemptive medical interventions. This risk-quantified management approach not only enhances the efficiency of healthcare resource allocation but also represents a significant shift from empirical practice to precision medicine in rabies vaccination management.

Stratified analysis revealed differential performance of the risk prediction model across patient subgroups, providing critical insights into its targeted applicability. Notably, patients with comorbid hypertension exhibited significantly elevated adverse reaction risk from high-risk exposure (OR = 4.43), likely attributable to pre-existing chronic inflammation and endothelial dysfunction that may amplify post-vaccination immune stress (52, 53). This observation is mechanistically plausible, as hypertension is characterized by a chronic low-grade inflammatory state and impaired endothelial barrier function. These conditions may predispose individuals to an exaggerated innate immune response following vaccination. The resultant pro-inflammatory milieu, involving elevated levels of cytokines such as IL-6 and TNF-*α*, coupled with a dysregulated endothelium that facilitates increased vascular permeability (54–56), could potentiate the local and systemic inflammatory cascade triggered by vaccine antigens. This pathophysiological synergy may explain the significantly higher risk of adverse reactions observed in hypertensive patients following the significant immune challenge of a high-risk rabies exposure. The model maintained stable predictive performance across age strata (<34 vs. ≥34 years), BMI categories, and seasonal variations, demonstrating broad clinical applicability. Notably, the significantly elevated risk associated with vaccination during summer and autumn (OR range: 2.11–3.74) warrants a mechanistic interpretation beyond a mere statistical association. The significantly elevated risk associated with summer and autumn vaccination (OR: 2.11–3.74) can be mechanistically explained by converging environmental and host factors. High ambient temperature and humidity may compromise vaccine thermal stability post-reconstitution (57, 58), while host factors like mild dehydration and thermal stress could prime a heightened inflammatory response (59). Behaviorally, increased outdoor activity with lighter clothing often leads to more severe animal injuries on exposed limbs, establishing a higher pro-inflammatory baseline. The synergy between vaccine instability, host physiological stress, and increased injury severity likely amplifies adverse reaction risk during warmer seasons. Interestingly, the association between high-risk exposure and adverse reactions did not reach statistical significance in the allergy history subgroup, potentially reflecting limited sample size or the dominant risk signal of atopic predisposition masking additional risk increments from exposure grading (60, 61).

These findings not only validate the model’s robustness but, more importantly, provide a nuanced risk stratification that enables individualized management. Critically, the strength and specificity of these associations warrant consideration of their potential causal nature. The observed associations, characterized by their strength, consistency, and specificity, offer evidence aligning with a potential causal role of vaccination in the adverse events documented in this study. The temporal sequence is clear, as all monitored events were defined and confirmed to occur following vaccine administration. The associations are biologically plausible. The robust link between bilateral deltoid administration and moderate-to-severe adverse events (OR = 4.73, 95% CI: 2.59–8.65, *p* < 0.001) is consistent with an antigen dose–response relationship, where increased antigenic load amplifies the local and systemic immune response (62). Similarly, the protective effect associated with greater skinfold thickness (OR = 0.935 per mm, 95% CI: 0.912–0.958, *p* < 0.001) coherently suggests a mechanism whereby subcutaneous tissue modulates the local pharmacokinetics and presentation of vaccine antigen, thereby attenuating the peak immunogenic stimulus (63). The strength of these associations is notable. Crucially, the stratified analyses (presented in full in the Supplementary material) reveal specificity in the risk profiles: procedural and anatomical factors (e.g., injection site, skinfold thickness) are primary drivers of local pain and event severity, whereas host-specific factors (e.g., allergy history) show a stronger, specific association with systemic fever (see respective result sections in the Supplementary material). This differentiation makes a generalized, non-specific confounding mechanism a less parsimonious explanation for the full pattern of results. While the observational design precludes definitive causal conclusions and unmeasured confounding cannot be entirely ruled out, the convergence of a firm temporal sequence, coherent biological mechanisms, strong effect magnitudes, and outcome-specific predictor profiles substantiates the interpretation that the identified factors are likely causally involved in the pathogenesis of post-vaccination reactions, advancing the interpretation beyond mere statistical correlation.

Collectively, the stratification results refine the model’s clinical application scope and enable truly individualized risk management through precise subgroup-specific recommendations. Several limitations of this investigation warrant consideration despite its contributions to risk prediction for rabies vaccination safety. The single-center retrospective observational design, while employing rigorous statistical methods to control for confounding, remains susceptible to inherent selection bias and unmeasured confounding. Furthermore, several methodological considerations should be noted. The primary outcome was a composite endpoint combining local and systemic reactions, which, while clinically pragmatic, may encompass biologically heterogeneous events. The extensive subgroup analyses presented are exploratory, and estimates derived from strata with limited sample sizes should be interpreted as hypothesis-generating rather than definitive, due to their inherent instability and increased risk of false discovery. To mitigate concerns regarding selection bias and assess the real-world performance of our model beyond the derivation setting, we applied the finalized nomogram to an independent, prospective cohort of 108 patients from the same institution. This external validation serves as a direct test of the model’s transportability and robustness to potential biases inherent in the original retrospective cohort. Although the sample size (*n* = 806) meets statistical requirements, the cohort from a single medical center may not fully represent the demographic diversity, animal exposure varieties, or clinical protocols across other regions, necessitating further validation of model generalizability through prospective multicenter studies. Specifically, key clinical decision determinants—such as the criteria for selecting bilateral deltoid vaccination—were not systematically documented in the medical records, creating a potential for confounding by indication that may not be fully captured by the measured variables.

In a preliminary effort to address this, we applied the finalized nomogram to an independent, prospective cohort of 108 patients from the same clinical setting. The model demonstrated encouraging transportability, with an area under the receiver operating characteristic curve (AUC) of 0.818 (95% CI: 0.724–0.911) and good calibration (Hosmer-Lemeshow test, *p* = 0.699). A specific consideration is the exclusion of patients receiving rabies immunoglobulin (RIG), consistent with standard prevention guidelines where RIG is typically reserved for Grade III exposures or immunocompromised individuals. While this may limit generalizability to RIG-treated populations—particularly high-risk exposures—it strengthens internal validity by eliminating confounding from RIG-related allergic reactions or immune interference, allowing clearer isolation of vaccine-specific and procedural risk factors. The study’s focus on the “Liaoning Chengda” rabies vaccine, which holds over 50% market share in China and is widely exported, enhances the practical relevance for this specific product, though model performance with other vaccine brands requires further investigation. Furthermore, while the model incorporates clinically accessible predictors, unmeasured confounders such as host genetic factors (e.g., HLA genotype), baseline immune status (e.g., specific cytokine levels), or detailed local inflammatory markers may affect prediction precision. Additionally, the absence of detailed data on certain clinical covariates, such as specific comorbidities beyond those captured, concomitant medication use, or detailed prior vaccination history, may represent residual confounding. While we controlled for major recorded comorbidities and allergy history, the influence of these unmeasured or incompletely measured factors cannot be fully ruled out and should be considered when interpreting the model’s estimates. Furthermore, it should be noted that adverse event grading followed the U.S. FDA Toxicity Grading Scale, a widely accepted standard in clinical vaccine trials. While the WHO and Brighton Collaboration frameworks provide alternative approaches, the FDA scale was selected for its granularity in severity assessment and its common use in regulatory contexts, facilitating comparison with other vaccine safety studies. The choice of this specific framework, along with the focus on recent and directly relevant literature, was made to ensure methodological consistency and clinical applicability, rather than to exclude other perspectives. Future studies integrating multi-omics data and refined immunological parameters could further optimize model accuracy and robustness.

In summary, this investigation successfully identified and validated key risk and protective factors for adverse reactions following rabies vaccination, developing a clinically applicable nomogram model with robust predictive performance that provides a valuable tool for advancing precision in vaccination safety management. Future research should focus on three strategic directions: first, validating the model’s generalizability and stability through prospective, multicenter, large-scale clinical cohorts; second, integrating immunomics and molecular biology techniques to elucidate key pathways and biomarkers underlying adverse reactions; and finally, developing mobile clinical decision-support tools (e.g., medical apps or hospital system plugins) incorporating the model to enable real-time risk calculation and intelligent early warning. These efforts collectively aim to advance rabies prevention practices toward enhanced precision and safety.

## Conclusion

This study establishes a clinically actionable framework for predicting and managing adverse reactions to rabies vaccination by integrating six evidence-based factors into an individualized nomogram model. The identification of modifiable risk elements (e.g., bilateral deltoid vaccination, delayed wound care) and protective features (e.g., 4-dose regimen, greater skinfold thickness) enables precise risk stratification and targeted interventions. While the model demonstrates robust performance and practical utility, its implementation in diverse clinical settings requires further validation through multicenter prospective studies and integration of molecular biomarkers. These findings advance rabies vaccination safety management from empirical practice toward precision prevention, offering a scalable tool for optimizing immunization protocols globally.

## Data Availability

The original contributions presented in the study are included in the article/Supplementary material, further inquiries can be directed to the corresponding author.
