# Supplementary material for: Risk factors for adverse events following rabies vaccination: a multivariate analysis of real-world data
Source: Front Public Health. 2026 Feb 4;14:1721524. doi: 10.3389/fpubh.2026.1721524 (PMC12913450; doi:10.3389/fpubh.2026.1721524)
Supplement: Supplementary file 1 [file Data_Sheet_1.PDF]

Table 1. Multivariable Logistic Regression Analysis for Predictors of Injection-Site Pain

	B	S.E.	Wald	P	OR	95%CI	
						Lower Limit (LL)	Upper Limit (UL)
Exposure Level	0.718	0.275	6.835	0.009	2.05	1.197	3.511
Injection Site							
Left					1.000		
Right	0.059	0.576	0.01	0.919	1.06	0.343	3.282
Both	0.86	0.375	5.269	0.022	2.363	1.134	4.925
Allergy History	1.573	0.397	15.688	<0.001	4.822	2.214	10.501
Wound Management Timing	0.83	0.362	5.271	0.022	2.293	1.129	4.658
Vaccination Regimen							
2-dose					1.000		
4-dose	-0.87	0.364	5.698	0.017	0.419	0.205	0.856
5-dose	-0.059	0.592	0.01	0.920	0.943	0.295	3.007
Skinfold Thickness at Injection Site	-0.024	0.015	2.605	0.106	0.976	0.949	1.005
Constant	-4.467	0.951	22.085	0.000	0.011		

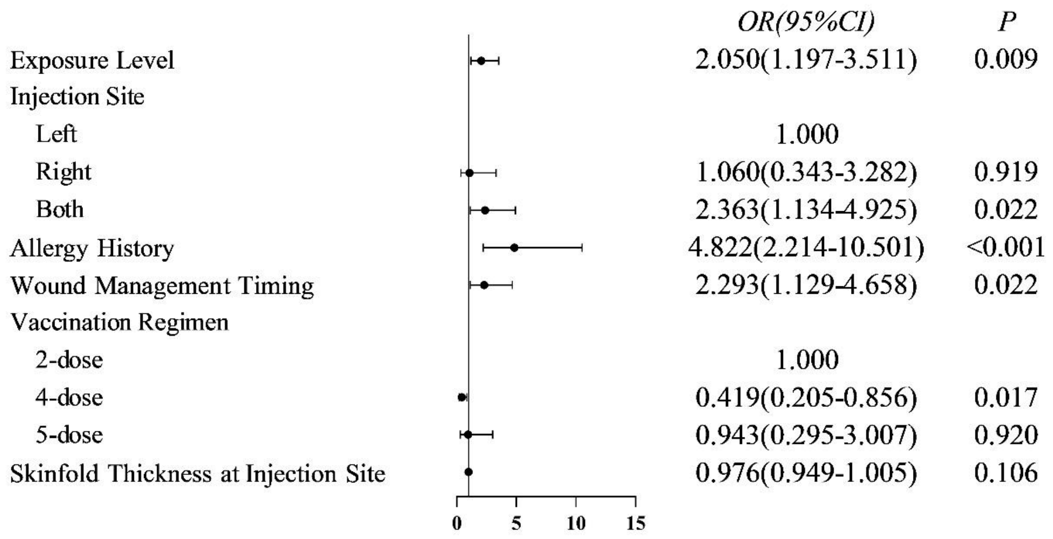

Figure 1. Forest Plot of Adjusted Associations Between Predictors and Risk of Injection-Site Pain

Table 2. Multivariable Logistic Regression Analysis for Predictors of Systemic Fever

	B	S.E.	Wald	P	OR	95%CI	
						Lower Limit (LL)	Upper Limit (UL)
Exposure Level	0.376	0.233	2.601	0.107	1.456	0.922	2.3
Injection Site							
Left					1.000		
Right	0.119	0.456	0.068	0.795	1.126	0.461	2.753
Both	1.329	0.318	17.468	<0.001	3.776	2.025	7.042
Allergy History	0.275	0.458	0.359	0.549	1.316	0.536	3.231
Wound Management Timing	0.037	0.262	0.02	0.887	1.038	0.621	1.736
Vaccination Regimen							

2-dose					1.000		
4-dose	-1.344	0.309	18.904	<0.001	0.261	0.142	0.478
5-dose	0.154	0.463	0.111	0.739	1.167	0.471	2.891
Skinfold Thickness at Injection Site	-0.057	0.013	19.481	<0.001	0.944	0.92	0.969
Constant	-1.054	0.696	2.293	0.13	0.349		

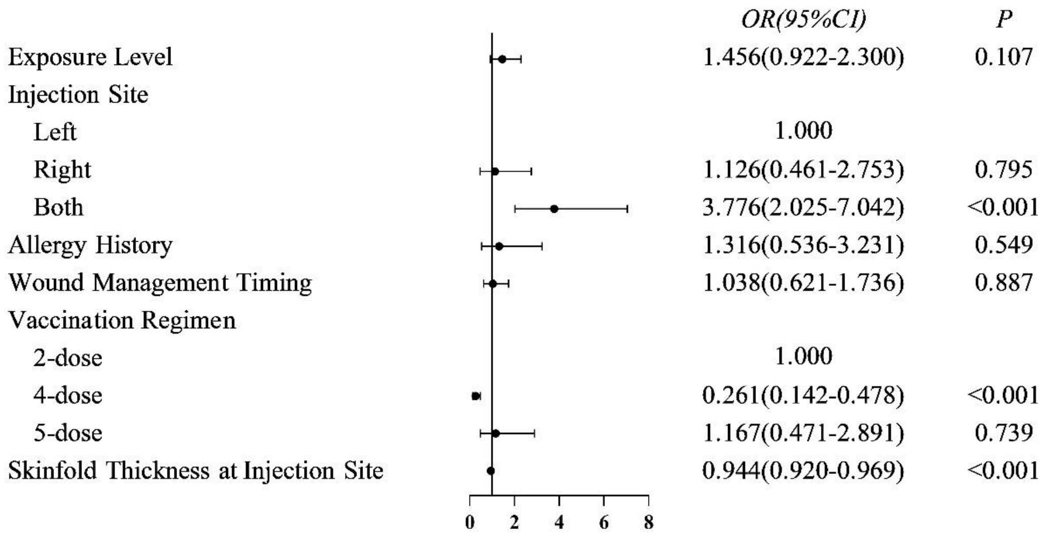

Figure 2. Forest Plot of Adjusted Associations Between Predictors and Risk of Systemic Fever

Table 3. Multivariable Logistic Regression Analysis for Predictors of Moderate-to-Severe (Grade ⩾2) Adverse Events

	B	S.E.	Wald	P	OR	95%CI	
						Lower Limit (LL)	Upper Limit (UL)
Exposure Level	0.414	0.22	3.537	0.06	1.512	0.983	2.327
Injection Site							
Left					1.000		
Right	-0.054	0.452	0.014	0.905	0.948	0.39	2.3
Both	1.554	0.308	25.471	<0.001	4.73	2.587	8.648
Allergy History	0.597	0.415	2.071	0.15	1.817	0.806	4.097
Wound Management Timing	-0.036	0.244	0.022	0.883	0.965	0.598	1.556
Vaccination Regimen							
2-dose					1.000		
4-dose	-1.554	0.3	26.901	<0.001	0.211	0.117	0.38
5-dose	-0.109	0.465	0.055	0.815	0.897	0.361	2.231
Skinfold Thickness at Injection Site	-0.067	0.012	29.125	<0.001	0.935	0.912	0.958
Constant	-0.507	0.649	0.611	0.434	0.602		

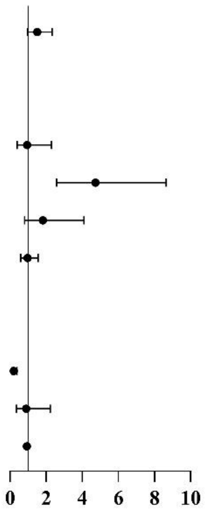

	OR(95%CI)	*P*
Exposure Level	1.512(0.983-2.327)	0.060
Injection Site		
Left	1.000	
Right	0.948(0.390-2.300)	0.905
Both	4.730(2.587-8.648)	<0.001
Allergy History	1.817(0.806-4.097)	0.150
Wound Management Timing	0.965(0.598-1.556)	0.883
Vaccination Regimen		
2-dose	1.000	
4-dose	0.211(0.117-0.380)	<0.001
5-dose	0.897(0.361-2.231)	0.815
Skinfold Thickness at Injection Site	0.935(0.912-0.958)	<0.001

Figure 3. Forest Plot of Adjusted Associations Between Predictors and Risk of Moderate-to-Severe (Grade $\geqslant 2$) Adverse Events
